# Where the joy comes from: a qualitative exploration of deep GP-patient relationships

**DOI:** 10.1186/s12875-023-02224-0

**Published:** 2023-12-13

**Authors:** Hayley Thomas, Johanna Lynch, Emily Burch, Megan Best, Lauren Ball, Elizabeth Sturgiss, Nancy Sturman

**Affiliations:** 1 General Practice Clinical Unit, Faculty of Medicine, The University of Queensland, Level 8, UQ Health Sciences Building (Building 16/901), Royal Brisbane and Women’s Hospital, Herston, QLD 4006 Australia; 2 Faculty of Health, Southern Cross University, Gold Coast, QLD Australia; 3 Institute for Ethics and Society, University of Notre Dame Australia, Sydney, NSW Australia; 4 Centre for Community Health and Wellbeing, The University of Queensland, Springfield, QLD Australia; 5 School of Primary and Allied Health Care, Monash University, Frankston, VIC Australia

**Keywords:** General practitioner-patient relationship, Primary care, General practice, Qualitative research, Attachment theory

## Abstract

**Background:**

Relationship-based, whole person care is foundational to quality general practice. Previous research has identified several characteristics of deep General Practitioner (GP)-patient relationships and their association with improved patient concordance, satisfaction and perceived health outcomes. Psychological attachment theory has been used to understand therapeutic relationships, but has only been explored to a limited extent in the general practice context. Additionally, evolving changes in sociocultural and commercial practice contexts may threaten relationship-based care. In view of this, we aimed to explore the nature and experience of deep GP-patient relationships, as identified by patients, from GP and patient perspectives.

**Methods:**

Semi-structured interview design. An initial survey assessed patients’ perceived depth of their relationship with their GP, using the Health Care Provider Attachment Figure Survey and Patient-Doctor Depth of Relationship Scale. Patients who reported a deep relationship, and their GPs, were purposively selected for individual interviews exploring their experience of these relationships. A post-interview survey assessed interviewees’ attachment styles, using the Modified and Brief Experiences in Close Relationships Scale. Patient interviewees also rated the patient-centredness of their GP’s clinic using the Person-Centred Primary Care Measure. Transcripts were analysed using thematic analysis.

**Results:**

Thirteen patients and five GPs were interviewed. Four themes characterised deep relationships: the ‘professional’; human connection; trust; and ‘above and beyond’. Patient, GP and practice team all contributed to their cultivation.

**Conclusions:**

We present a revised conceptual framework of deep GP-patient relationships. Deep relationships come to the fore in times of patient trouble. Like attachment relationships, they provide a sense of safety, caring and support for patients experiencing vulnerability. They can stretch GP boundaries and capacity for self-care, but also provide joy and vocational satisfaction. Patients may not always desire or need deep relationships with their GP. However, findings highlight the importance of enabling and cultivating these for times of patient hardship, and challenges of doing so within current healthcare climates.

**Supplementary Information:**

The online version contains supplementary material available at 10.1186/s12875-023-02224-0.

## Background

Relationship-based, whole person care is foundational to quality general practice. [1, 2] Trusting doctor-patient relationships improve patient concordance, satisfaction and perceived health outcomes. [2–7] However, changing sociocultural and commercial practice contexts may threaten relationship-based care. [3, 8] It is important to understand the character and cultivation of deep GP-patient relationships to maintain their benefits.

Previous literature has identified ongoing depth of doctor-patient relationship as important to patients. [9, 10] Synthesis of qualitative literature on patients’ perspectives found that deep patient-doctor relationships were characterised by knowledge, trust, loyalty and regard [9]. Similarly, a study exploring ‘healing relationships’ in primary care involved interviews with GPs considered by the authors to be ‘exemplar healers’, and patients whom these GPs selected. [11] This study identified that trust, hope and a sense of being known characterise healing relationships, and that such relationships are fostered through GPs valuing patients, appreciating power and abiding. In other work, building a whole person ‘sense of safety’ in patients and clinicians has been identified as an important therapeutic goal for primary care relationships. [12] Multiple factors influence GP-patient relationships, including doctors’ relational skills and attitudes; practice characteristics; health systems and demographics. [13, 14].

The psychotherapy relational model of attachment theory may have relevance to GP-patient relationships. [10] Originally studied in parent-child relationships, and then in adult intimate relationships, attachment theory has been applied to healthcare relationships. [10, 15, 16] Attachment theory proposes that humans require connection in safe relationships for health. [17] Attachment figures provide both a ‘safe haven’ to offer comfort and a ‘secure base’ to support exploration. [18] Each person’s connection, or attachment, style (secure, anxious or avoidant), is influenced by early childhood experience [10, 16]; availability of the attachment figure to be wiser, kinder, and stronger; and attunement within the relationship. [18] Attachment figures are not easily replaced and can influence capacity to trust and trigger strong emotions, proximity seeking and separation protest. [18] Healthcare studies have suggested that doctors may serve as attachment figures for patients, providing a sense of safety amidst healthcare-related vulnerability. [10, 15, 16] However, current moves toward commercialised medicine and systems that reduce direct GP-patient contact may threaten the interpersonal continuity underpinning such relationships. [8, 10] Additionally, it has been suggested that attachment theory is of limited relevance to understanding doctors’ motivation for caring. [16].

This project aims to build upon this background by further characterising the nature of deep GP-patient relationships and exploring how these are cultivated, from GP and patient perspectives.

## Methods

**Fig. 1:**
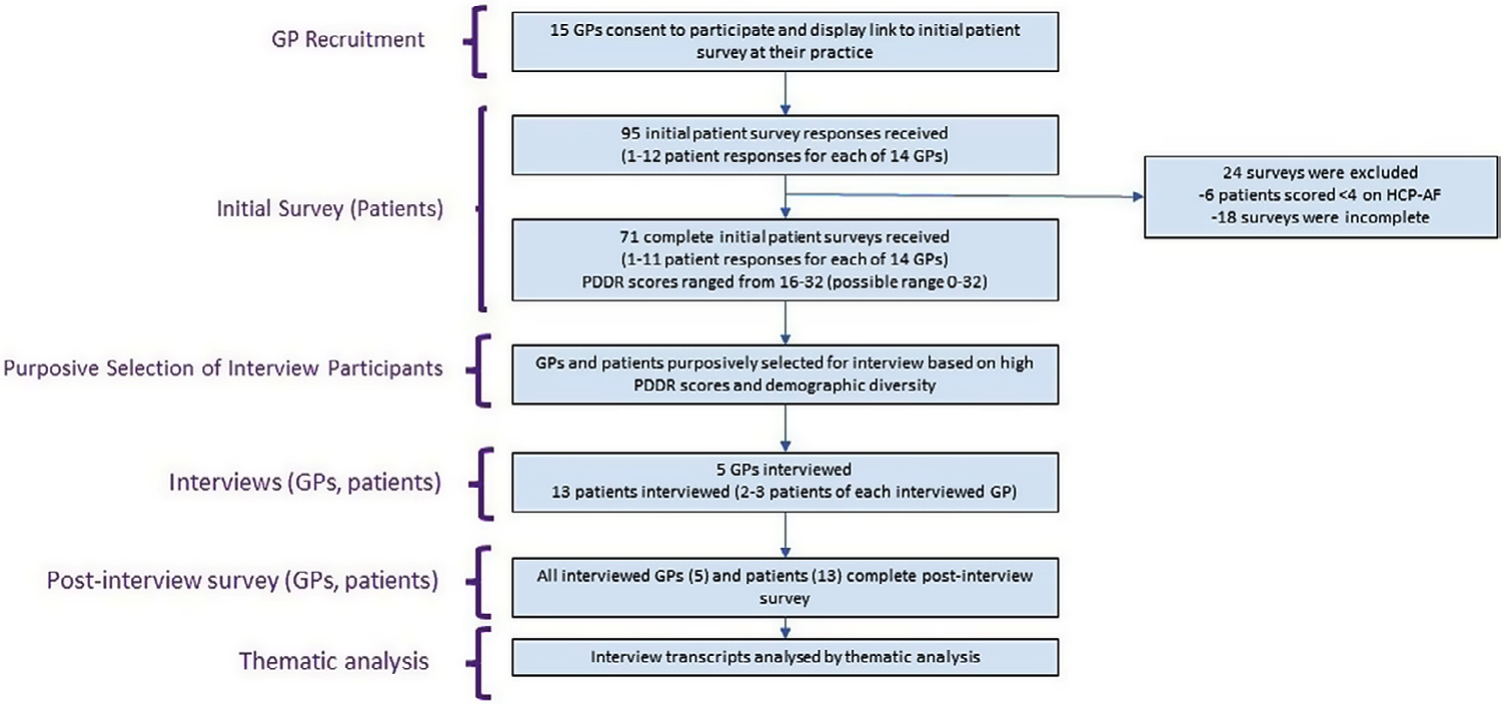
Study design and participant flow

Semi-structured interview design, with purposive selection of GP-patient pairs with deep relationships, as identified from an initial patient survey (Fig. 1).

### Setting

Australian General Practice. General practice forms the foundation of Australia’s health system and operates on a fee-for-service model. Patients are not required to register with GP practices. The patient fee is subsidised by the government-funded Medicare scheme, and either accepted as full payment (bulk-billing), or supplemented by a patient co-payment (mixed or private billing). [19].

### Research team background

HT, JL, ES and NS are GPs who combine clinical and academic practice. MB is a palliative care physician and ethicist with a background in qualitative methodologies. EB and LB are primary care researchers with a background in dietetics.

### Participants and recruitment

English speaking GPs and GP registrars practising in Australia and their adult patients were eligible to participate. Study information was emailed to general practices affiliated with The University of Queensland, practices identified online and researchers’ personal contacts, and followed up with a phone call. The study was advertised in GP newsletters and social media. Participating GPs and their practice manger or principal provided written informed consent.

Patients of participating GPs were invited through waiting room flyers to complete an online survey (Qualtrics XM, Supplement 1) [20] including demographic information and validated doctor-patient relationship scales (Health Care Provider Attachment Figure Survey (HCP-AF) [15]; Patient-Doctor Depth of Relationship (PDDR) scale). [21] The HCP-AF assesses whether a health care provider may serve an attachment function for a patient, and comprises 5 ‘yes/no’ questions (1 point for each ‘yes’ answer’); its median score in an online sample has been reported as 5. [22] Study participants who scored 4 or above on the HCP-AF (indicating a possible attachment relationship with their doctor) completed the PDDR. The PDDR is a validated 8-item scale that was developed following a review of qualitative literature reporting patients’ perspectives on doctor-patient relationships, and measures patient-doctor depth of relationship. [9, 21] Items are scored from 0 to 4 and, if all questions are completed, item scores are summed to give a total score ranging from 0 (low) to 32 (high). [21] The median score in a clinical sample has been reported as 26, and deep relationships defined by scores of 31 or 32. [21].

Patients with HCP-AF scores of 4 or above and high PDDR scores were purposively selected for diversity of age, gender, relationship duration, and presence/absence of a chronic health condition. Selected patients and their GPs were invited to participate in a semi-structured interview.

### Semi-structured interviews

Interviews were conducted via telephone or Zoom [23] video-conference. HT conducted GP interviews and EB conducted patient interviews, both to align researcher and participant backgrounds to facilitate information gathering, and to eliminate risk of unintentionally compromising confidentiality with either member of the GP-patient pair during interviews. Interviews explored participant experiences of GP-patient relationships (Supplement 2) and were recorded and transcribed using a professional transcription service.

### Post-interview surveys

Interview participants completed an online (Qualtrics XM, Supplement 3) [20] post-interview survey assessing their attachment style [24], demographics (for GPs) and perceived person-centredness of the GP practice (for patients). [25].

Attachment style was assessed using an adapted version of the modified and brief Experiences in Close Relationships Scale (ECR-M16). [26] The ECR-M16 is validated for use in medical settings and includes two subscales (attachment anxiety, attachment avoidance). Each subscale comprises eight items, rated from 1 (low) to 7 (high), which are averaged to give an overall score for attachment anxiety and avoidance. A normative range was not reported in Lo’s original ECR-M16 validation study [24]; the authors therefore searched Scopus on 9 November 2023 for all citations of Lo’s study and identified those reporting English language ECR-M16 results in primary care or population samples. Average scores for anxiety and avoidance ranged from 2.6 (SD 1.0) to 3.0 (SD 1.3) and 2.2 (SD 1.0) to 3.1 (SD not reported) respectively. [22, 26, 27] Therefore, GP and patient participants who scored less than 2.6 on the anxiety scale, or 2.2 on the avoidance scale, were considered ‘low’ anxiety or avoidance respectively, and those who scored above 3.0 on the anxiety scale, or 3.1 on the avoidance scale, were considered ‘high’ anxiety or avoidance respectively.

Patients’ perceived person-centredness of their GP practice was measured using the validated Person-Centred Primary Care Measure (PCPCM). This comprises 11 items, rated from 1 (low) to 4 (high), whose scores are averaged to give the final PCPCM score. The average PCPCM score in a clinical primary care sample was 3.5. [25] Therefore, scores below 3.5 are considered ‘low’ and scores above 3.5 are considered ‘high’ in this study.

### Data analysis

Interview transcripts were analysed using inductive thematic analysis with NVivo Pro software, looking for themes describing the nature and cultivation of deep GP-patient relationships. [28, 29] Six transcripts were coded by multiple authors, with consensus reached by discussion. HT coded remaining transcripts and all authors determined themes by discussion, informed by previous frameworks regarding healing relationships and attachment theory. [9–11, 30, 15, 18, 31] This involved iterative and reflexive processes, including rereading transcripts and several meetings to discuss themes as they emerged. HT also compared themes between high vs. low attachment anxiety and avoidance groups (assisted by NVivo Pro matrix coding queries), looking for any obvious inter-group differences; and coded transcripts deductively for characteristics of attachment relationships (including safe haven, secure base, availability, stronger/wiser, strong emotions, particularity, proximity seeking, and mental representation). [18].

## Results

### Participant characteristics

Ninety-five initial patient survey responses were received and seventy-one of these included complete PDDR scores (Fig. 1). PDDR scores ranged from 16 to 32 (mean 27.6, SD 4.5).

Thirteen patients with high PDDR scores (30–32), and their five GPs (two to three patients per GP), were selected for interview (Table 1). Female patients were over-represented in this sample (69%), this likely in part reflects more GP visits among Australian females (57% of total GP attendances in 2021-22) [32], and in part difficulty recruiting males to participate in the study. The sample included two males with a PDDR score of 30 (below the cut-off of 31 for deep relationships in Ridd’s study) [21] to obtain demographic diversity.

### Post-interview survey results

GP interview participants’ average attachment anxiety score was low (2.25), and average attachment avoidance score was within the range of published means (3.1). Three GPs were classified as low anxiety, one as high anxiety, one as low avoidance, and three as high avoidance. Patient interview participants’ average attachment anxiety score was high (3.4) and average attachment avoidance score was within the range of published means (2.75). Three patients were classified as low anxiety, eight as high anxiety, four as low avoidance, and five as high avoidance. No relationship was evident between the attachment orientation of GPs and those of their patients.

Eleven patient interview participants scored their GP practice highly on the PCPCM. The remaining two participants gave their GP practice a low score; these two participants attended the same practice.

**Table 1:** Interview participant characteristics

	GP Participants	Patient Participants
Number	5	13
Gender	Female: 3 (60%) Male: 2 (40%) Non-binary: 0 (0%)	Female: 9 (69%) Male: 4 (31%) Non-binary: 0 (0%)
Age	30-49yrs: 2 (40%) 40-49yrs: 1 (20%) 60-69yrs: 1 (20%) Not stated: 1 (20%)	18-24yrs: 1 (8%) 25-34yrs: 2 (15%) 35-44yrs: 1 (8%) 45-54yrs: 1 (8%) 55-64yrs: 1 (8%) 65-74yrs: 3 (23%) 75-84yrs: 3 (23%) > 84yrs: 1 (8%)
Time Practising as a GP	5-10yrs: 2 (40%) 10-20yrs: 1 (20%) > 20yrs: 2 (40%)	N/A
Sessions (half days) worked per week	5–6 sessions: 1 (20%) 7–8 sessions: 1 (20%) 9–10 sessions: 2 (40%) > 10 sessions: 1 (20%)	N/A
Practice Billing Model	Bulk Billing: 1 (20%) Private Billing: 2 (40%) Mixed Billing: 2 (40%)	N/A
Practice Suburb Index of Relative Socioeconomic Advantage and Disadvantage (1 lowest socioeconomic status, 10 highest) [33]	1: 1 (20%) 8: 1 (20%) 10: 3 (60%)	1: 2 (15%) 8: 3 (23%) 10: 8 (62%)
Chronic Illness	N/A	Yes: 9 (69%) No: 4 (31%)
Duration of GP-Patient Relationship	N/A	< 1 year: 1 (8%) 1–2 year: 2 (15%) 2–5 year: 2 (15%) 5−10 year: 1 (8%) 10–20 year: 4 (31%) > 20 year: 3 (23%)
Number of Visits to GP in Previous 12mnths	N/A	1–2 visits: 1 (8%) 3–7 visits: 5 (38%) 7–10 visits: 2 (15%) > 10 visits: 5 (38%)
HCP-AF Score (mean, range)	N/A	5, 5–5
PDDR Score (mean, range)	N/A	31.6 (30–32)
PCPCM Score (mean, range)	N/A	3.7 (2.8-4.0)
ECR-M16 score (mean, range)	Anxiety:2.25 (1-3.8) Avoidance: 3.1 (1-3.9)	Anxiety: 3.4 (1.5–5.6) Avoidance: 2.75 (1-4.75)

### Interview themes

**Fig. 2:**
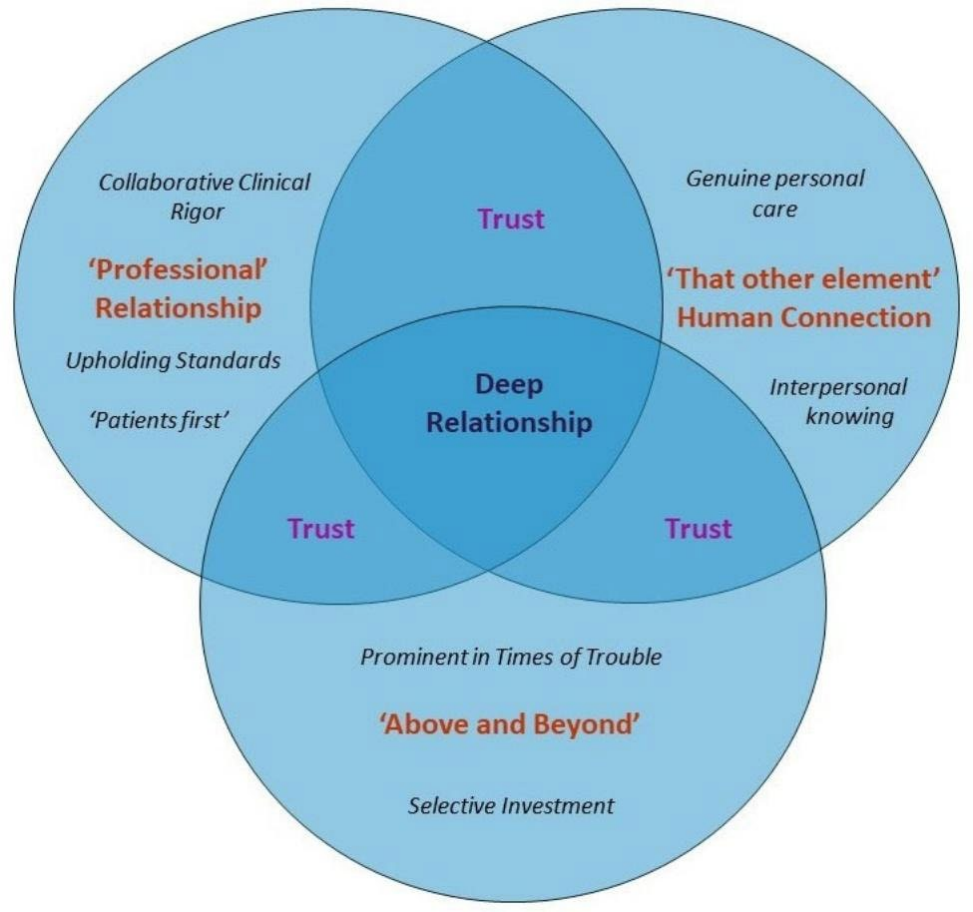
A model of deep GP-patient relationships. Deep GP-patient relationships intertwine professional dimensions with ‘that other element’ of human connection and are permeated by trust. They are fostered by GPs going ‘above and beyond’ expectations, particularly in times of patient difficulty

Four themes describing the nature and cultivation of deep GP-patient relationships were identified: the ‘professional’; the ‘other element’ of human connection; trust; and ‘above and beyond’ (Table 2; Fig. 2). Where relationship between attachment styles and thematic content were evident, these are noted under the relevant themes below.

**Table 2:** Themes

Theme	Subthemes
‘Professional’	Collaborative clinical rigour Upholding standards ‘Patients first’
‘That other element’: Human connection	Genuine personal care Interpersonal knowing
Trust	
‘Above and beyond’	Prominent in times of trouble Selective investment

### Theme one: ‘professional’

GPs and patients described GP-patient relationships as ‘*professional’* (GP1-2, GP5, P11-13). This term denoted clinical knowledge and rigour, and upholding standards of good practice and presentation. GPs also discussed ‘putting the patient first’.

### Collaborative clinical rigour

Patients reported that GPs’ clinical competence was a key priority. They appreciated clinical knowledge and a thorough approach.

One patient commented

*‘…the way [GP] looks at it is much more of a holistic…picture than I have seen from some GPs in the past who…are happy to…prescribe…tablets and send you on your way’* (P6).

However, GPs commented that not all patients sought this thorough approach:

*‘I’m very thorough, or long winded, depends on how you feel…not every single patient will like my style…’* (GP1).

Patients commented that their GP was ‘*knowledgeable’* (P4, P5). Both GPs and patients were aware of a knowledge differential:

*‘I have a medical degree and medical knowledge, and most of my patients do not’* (GP3); *‘I’m not qualified to have a difference of opinion with [GP name]…I’m afraid I put my complete trust in him’* (P9).

However, patients were often also aware of GPs’ fallibility, and most played an active role in their own healthcare decisions. Several GPs deliberately facilitated this:

*‘…you’ve both got a problem to solve, so you work at the problem together and find a solution’* (GP5).

They sought to minimise the doctor-patient power imbalance:

*‘I really try to engage with [patients]…as equals…it’s important for patients to feel like they’ve got some choice about…the treatments I suggest…I just try to be really open with patients and…not have a power differential…making the patient feel like they’re on a team with you’ (GP4).*

They also described actively listening to patients’ agendas and ideas; communicating information honestly and accessibly; providing patients with choice; empowering them to *‘take control of their health’* (GP3); and when necessary, accepting patients’ prerogative not to accept their advice.

### Upholding standards

Patient and GP participants expected GPs to adhere to standards of good medical practice. Some GPs contrasted this with consumerist relationships, in which patients were entitled to their preferred treatment due to paying for GP services:

*‘… [some patients] think of you like a tradesman…Put on a new roof or do that…they think it’s that sort of relationship. No, a professional relationship isn’t like that’* (GP5).

For example, GPs declined requests for inappropriate treatment (e.g., unnecessary antibiotics (GP2, P1)):

*‘I don’t have much control of what people want…but I will try to…preserve my traditional professional way’* (GP1).

Some patients valued this:

*‘…I’ve raised my eyebrows after attending [a different GP]…once I went and I…had a virus…and he gave me antibiotics…he said, “some people like taking something”…I just looked at him, I grabbed the script and I…ripped it up in front of him and…said, “That’s not what you do”’ (P1).*

Additionally, some GPs and patients identified presentation and the physical practice environment as important:

*‘I always wear long sleeves shirt, tie, trousers…it just gives you a bit of respect. If you have neat and tidy rooms…[patients] say, “Oh, we’re seeing the doctor”’* (GP5).

Patients commented on their GP’s,

*‘beautiful medical [clinic]…you could eat off [the] lavatory that’s how clean it is…’* (P3).

### Patients first

GPs expressed a sense of professional responsibility, sometimes at personal cost. In difficult patient interactions, one GP identified responsibility to *‘realise your professionalism and that you’re there to add value’* reflecting that,

*‘…[it] could be to my detriment, but I always put the patient first’* (GP5).

Some GPs reflected that conveying care sometimes required masking their feelings:

*‘I try to avoid [appearing stiff, bored or unhappy] and seem like I’m happy to see them and…take on the challenge of helping them…even if I’m not feeling that way that day’* (GP4).

GPs attached moral significance *to ‘do[ing] a good job’* (GP1, GP5) and some described their role as more than a job:

*‘…to me, general practice is…a vocation’* (GP5).

All recounted situations when this was personally costly, entailing missed breaks, out-of-hours work, reduced income or internal distress at balancing competing demands. One reflected:

*‘…being a Mum and a doctor is not an easy thing to do…I’ve still got to get to school pickups…swimming…cook and clean and walk the dog and…spend time with the children and do the homework…and then somewhere in there we’re meant to be finding time for self-care as well’ (GP2).*

Notably, however, GPs with low attachment anxiety seemed to accept that they would not be able to please all patients, and reflected that this was protective for their wellbeing:

*‘…if I took an approach to the job where I was…stressing about [patients] or worrying too much about what they thought of me, I guess that might be harder.’ (GP4)*.

Patients recognised that a GP’s

*‘job…is…not easy’* (P11).

Some expressed tolerance of their GP’s humanity:

*‘…everybody has their ups and downs…’* (P3).

They reported responding graciously when their GP ran late:

*‘…all doctors’…situations are different and they run over time and…there’s emergencies that come through, and I’m extremely flexible with that kind of thing…’* (P4).

Some reported only seeking urgent on-the-day care when they believed it was truly necessary and accepting that they may need to wait for an appointment or see another doctor if their GP was on leave or unavailable.

Both GPs and patients described experiences of relationship breakdown with previous GPs or patients. Dissatisfied patients reported having sought care elsewhere, and GPs offered to transfer patients’ care elsewhere when the

*‘therapeutic relationship [had] broken down’* (GP5).

One GP appreciated patients being,

*‘willing to let me know when they’re dissatisfied with something that I’ve done or said, or a referral that I’ve made…that to me is a really…big value-add’* (GP2).

### Theme two: ‘That other element’: human connection

The second theme we term ‘*that other element’* (P1), to capture non-medical aspects that patients valued as much as the ‘professional’ relationship. One patient commented,

*‘medical knowledge and stuff like that…is very important…but also too is that other element’* (P1).

‘That other element’ involved human connection, characterised by genuine personal care and interpersonal knowing.

### Genuine personal care

While GPs tended to emphasise the importance of being respected as a professional, patients valued a sense of genuine personal care. Participants identified GP actions that communicated this care (Table 3).

**Table 3:** GP actions communicating personal care

Action	Quotations
Demonstrating humility and non-judgemental respect	‘…within all professions…people…see themselves as a little bit better…what I would like to see in…any profession, medical or otherwise, that everybody is given the respect they deserve because they’re a human being as well…and that’s what [GP name] does… I was down [at the GP clinic] one day…[these] young [people] walked in…they were looking very awkward…you could see that people were looking at them, they had lots of tattoos, they weren’t dressed particularly well at all…but straight away, [the GP] said, “Oh please have a seat here”…like, welcome to the clinic you are here the same as everybody else…myself and my staff are here to help you.’ (P1). ‘When [my GP] comes out [they’ll] smile at me and [they’ll] say “Hey…I won’t be long” …[they] always acknowledges me when I’m in the waiting room and…I really like that…I’ve even bumped into [my GP] outside of the surgery…at dinner one time and…we both waved at each other and said hello and… [they weren’t] like trying to hide [their] head or pretend that I wasn’t there’ (P4). ‘…there were quite a few times where I felt like [other GPs] were…quite judgemental or offering advice that didn’t seem helpful…and comments about my weight’ (P6).
Showing interest in patients as people (i.e., in non-medical aspects of patients’ lives)	‘…before [they start]…talking [they’re] like, “Oh, how you been, how are the children?” …[they’ll] remember things that you’ve previously said…it never feels like you’re coming in there and you’re sitting down and [they’re] straight to… “What are you here for?”, so I really like that because…I feel like it develops a rapport between the doctor and the patient.’ (P4)
Taking time to actively listen	‘…it’s always worth [waiting to see my GP] because [they do] take the time to speak with you and…to listen to what you have to say…[they’re] not in a hurry to push you out the door’ (P7) ‘…you enter [the GP’s] rooms and…[the GP is] totally focussed on you, [they’re] not thinking about I’ve got four or five other patients out in the waiting room…[GP name] puts in the time…you walk in there and you’re [their] only patient until we finish dealing with what we’ve got to deal with.’ (P8)
‘Above and beyond’ (P1)	‘…when my Dad passed away, they actually sent flowers on the day of the funeral… [my GP] is above and beyond…’ (P1) ‘…[my GP] said, “It’s alright…I haven’t got an appointment after you, it’s my lunch break”…[they were]…going to sacrifice some of [their] lunch time to spend time with me on this problem…that made me feel pretty good about [them]…[they] seemed like [they were]…a genuine person…’ (P10) ‘…where [my GP is] really wanting me to go and see somebody quite quickly [they’ll] organise it for me rather than just give me the details and make me organise it myself, like [they’ll] actually call up the surgery and say, “I have a patient here I really would like them to see the doctor ASAP…And [they’ll] really help to make that happen…I really like that.’ (P4) ‘I felt like [the GP] went the extra mile because [they] really cared and [they] knew me and …we’d been able to develop that little bit of relationship…it was really nice to have somebody that actually just went in to bat for you rather than just went, okay, I’ve got your results back, you need to have an iron infusion, organise that for you when you get back…[they were] genuinely concerned about my welfare and that made me really put [them] in a high regard as to a…really decent GP that actually cares about the patient.’ (P4) ‘[I told my GP about domestic violence]…so then we kind of just used some of that…appointment…and [they were]…Googling…DV services because [they] couldn’t remember who the ones that they…had worked with and [they]…gave me all their information and their phone numbers and things that I potentially needed to do…I suppose it’s still kind of within [their] scope but…I don’t know if that’s just like standard care…it just felt like it was very thorough’ (P5) ‘And [patient’s will] say, “Do you remember the day,” …sometimes I’ll take them to school, I’ll pick them up from their home and take them to school. I’ve done all that sort of thing before if I think it’s appropriate. And if their parents are having a really hard time. They say, “Do you remember the day…you took me to school” …that was a key thing in their minds. You know, for me that was just one of many.’ (GP5).

GPs expressed genuine respect for patients:

*‘I have a tremendous amount of respect for [the patient]’* (GP2).

They derived a sense of satisfaction, purpose and value from providing relationship-based care:

*‘I really love to see things grow and develop. I love seeing…people doing well…it’s highly motivational and it’s so addictive…you learn so much every day…Where does the joy come from?…the joy comes from relationships in general practice …and doing a good job’ (GP5).*

GPs acknowledged that cultivating GP-patient relationship,

*‘is a really big part of what our actual job is…the stuff that’s actually hard and…worth putting effort into’* (GP4).

They believed this was protective:

*‘…it makes avoiding burnout easier…feeling that you’re appreciated and feeling a sense of…continuity and purpose in your work…And…having regular patients that you have a…good trusting relationship with…’ (GP4).*

A sense of genuine care influenced whether patients continued the relationship with their GP:

*‘I felt like, oh, wow, I really like this [GP]…I want to continue with [them] because…I feel like [they] actually care…about me’* (P4).

Patients contrasted their GP’s care with primarily consumerist relationships and previous depersonalising and isolating experiences:

*‘…[the GP] actually care[s] about you as a person, not just as a person bringing money into their clinic or…business to their doorstep…’* (P13); *‘…[GP name] talks to you like you’re a person you’re not just a…number…I’ve been to doctors where you’re just a number’* (P10); *‘…I felt really isolated, and I felt not listened to, and I felt like [the GP] was judging me…’* (P4); *‘…you walk out and you feel as though…what do I matter?’* (P1).

These reports of feeling dismissed or judged by previous GPs tended to feature more prominently in interviews with patient participants with high attachment anxiety scores.

While participants referenced a fit between GP and patient personalities, *‘shared interests’* (P11) and shared humour, GPs and most patients distinguished GP-patient relationships from friendships. One GP reflected:

*‘I think you can have a very friendly relationship but…[GPs] who think they can be friends to their patients [are]…kidding themselves…You have a power imbalance…in the consulting room that is a different relationship…a very precious relationship that we need to foster and develop…’* (GP5).

Patients described the relationship as *‘like a friendship [or ‘mate’]*’ (P4, P6, P10). They nonetheless retained some distinction:

*‘…it’s always been professional, as friendly as it is.*’ (P11).

### Interpersonal knowing

GP and patient participants described a mutual (though asymmetrical) interpersonal knowing, developed within the longitudinal, multigenerational, community general practice context.

One GP reflected that their patient *‘saw me grow’* as a GP from early in their career (GP1), and patients reflected that their GPs’ approach could evolve over time:

*‘…in the past [the GP] would just say what [they] thought I should do…but now if I…make a suggestion…[they] will say, “Well, yeah, that’s a good idea.” Or “No, I don’t think that’s a good idea”’ (P12).*

Patients and GPs also reported growing to know each other as people: a patient reflected:

*‘…we’ve gone through a lot…I’ve got to know [them] better and [they’ve] got to know me better…I won’t go into [their] personal life, but [they] went through something very devastating…you get to know the person’ (P11).*

Consistent with this, GPs reported judicious self-disclosure on occasions to assist patients:

*‘…in some instances when you have a patient who’s experiencing something [similar] to your own experiences…there’s an opportunity to say…when I was that age I…had trouble with something similar…I remember how tough it was…but [I] probably won’t open up about my deepest and darkest’ (GP2).*

GPs also often demonstrated considerable knowledge of their patients’ personalities, and family and social contexts, as well as their medical histories:

*‘…there’s times I’ve gone in and seen [my GP], and [they saw] straight away that I’m not feeling 100%…’* (P8).

GPs talked about personalising their care, adjusting according to perceived patient preference:

*‘…patients…want different styles…some patients…say, “Doc, tell me what to do…” And others…want to know every detail…you have to be responsive to different people’s needs’ (GP5).*

Patients had a role to seek continuity with their GPs to support this ongoing relationship.

### Theme three: trust

Trust was a strong theme which permeated both the ‘professional’ and the human dimensions of deep GP-patient relationships.

Both patients and GPs emphasised the critical importance of trust. One GP stated,

*‘…I think that the value that…we give patients as a general practitioner is…being…someone that they trust…’* (GP4).

A patient reflected,

*‘I trust [my GP] …I trust [them] 100%’* (P9).

This was earned over time:

*‘…I trust [my GP] implicitly…with my health…[they’ve] earnt that trust, it’s not something that I…throw out there…’* (P8).

Trust was mutual; a GP reflected:

*‘…when you know the patient, you’ve got some mutual trust and rapport…some of your planning around safety netting and things like that becomes a lot easier…you believe that they’re going to follow something up if you’ve stressed it…’ (GP4).*

Likewise, a patient felt,

*‘… [my GP] completely trusts in me and my ability to…relay…information rather than making me feel like I’m a hypochondriac…’* (P4).

Patient trust extended to the GP’s practice and other healthcare professionals whom they had recommended.

Trust was closely linked to patients’ sense of safety and comfort:

*‘I feel very safe in any questions I have to ask [my GP] …[I] feel safe and really able to be candid with [them]’* (P13).

One patient reported that thinking about their GPs’ advice comforted them amidst mental health challenges:

*‘…when things were getting bad, I’d just relax and think about what [GP name] said…*’ (P10).

Another patient reported a sense of security, believing their GP would assist if required:

*‘I live on my own…as you get older, you’re quite vulnerable and things go wrong…it’s very reassuring to have that backup of somebody who knows you and knows that you need help, and they will give you help’ (P11).*

GPs also provided patients with a secure reference point within a complex healthcare system:

*‘… [GP name] has been my support the whole time…even when I was thrown from hospital to hospital because they did not know what to do with me.’* (P1)

Several patients expressed concern about what they would do when their GP retired:

*‘…the only fear I have at the moment is…how long is he going to continue practicing and if he leaves, who would I see?*’ (P12).

Some patients linked trust with taking GPs’ advice:

*‘If [my GP] tells me to do something, I do it because I know it’s for my own good…I sort of pay it back that way…’* (P10).

However, one patient recounted that:

*‘… [my GP] was asking me how I was going with the supplements I’m meant to be taking…I was like… “Ooh, sorry”…’* (P5).

### Theme four: ‘above and beyond’

Depth in the GP-patient relationship was not always desired by patients, and GPs did not always invest equally in cultivating deep GP-patient relationships.

### Prominent in times of trouble

GPs and patients believed that deep GP-patient relationships were most valued in times of patient difficulty. One GP described this as ‘grand final day’:

*‘On grand final day you have no control…You develop a relationship and when you say…“There’s a lump in your breast, we need to investigate that” they take it seriously, you know?’* (GP5)

Patients appeared to value deep GP-patient relationships less when they required only intermittent care for minor ailments:

*‘I guess I didn’t put too much value in it…not didn’t value it, but…it was just always…small things…I didn’t think…continuity of care was…an important thing…’* (P5).

Conversely, they tended to seek deeper relationship during times of chronic illness, health crises, mental health struggles or personal difficulties:

*‘If it’s anything really urgent or serious or personal, I will wait…for [GP name], probably, but…I’ve seen many other doctors in the practice and…I have no problem with that at all’* (P11).

One patient who had *‘battled with suicide for the best part of…10 years…’* recounted,

*‘…the fact that I’m still here today…I would attribute in part to [the GP] and the work [they] put in…’* (P8).

Several patients described their GPs going *‘above and beyond’* (P1) expectations to assist them in a crisis. Examples included urgent care provision, active follow up, health advocacy and sending flowers; and a GP even mentioned offering assistance with dropping children to school.

#### Selective investment

GPs invested more in cultivating patient relationships when starting a new practice, or for new or complex patients who were likely to remain under their care, rather than with patients who were seeing multiple doctors.

Time availability also affected GPs relational cultivation:

*‘…the last six months have been incredibly busy, so I don’t think my relationship building has been awesome of late.’* (GP2).

Patients were aware that GPs needed time to be able to offer personalised care, and that this often came at a cost:

*‘…the fact that it’s not a bulk billing practice…does allow for…more…personalised service…it sounds shitty that you have to…pay for a good doctor…not [that]…the…other ones aren’t good doctors, they probably just aren’t afforded…the same amount of…time…’* (P5).

Other practice staff also differed in their investment in deep relationships. Patients emphasised the importance of practice staff exhibiting the same genuine personal care, availability and professional approach as the GP, in contrast to a *‘production line’* (P1) approach. Interviews suggested variable cohesion between the GP and practice staff: one GP described regular team communication and a strong sense of cohesion; while another described providing patients with strategies to bypass reception staff to secure an appointment for urgent care.

## Discussion

Findings suggest that deep GP-patient relationships are characterised by intertwining ‘professional’ aspects (collaborative clinical rigour, upholding standards, ‘patients first’) with ‘that other element’ of human connection, comprising genuine personal care and interpersonal knowing. Trust seems to permeate these relationships, which tend to come to the fore in times of difficulty. GPs typically invest time and effort early in these relationships, although they grow over time as patients and GPs get to know each other as people.

Findings are consistent with previous research that trusting GP-patient relationships facilitate continuity of care, enable the GP to provide effective motivation and reassurance and improve treatment concordance. [2–7] They align with previous studies, using different recruitment strategies, that also identified personal and professional aspects of the GP-patient relationship; the importance of interpersonal knowledge, mutual trust and balancing power; and the sense of safety existing in such relationships. [2, 9, 11, 12].

Findings support previous proposals that GP-patient relationships serve attachment functions for patients as a ‘safe haven’ and ‘secure base’ when they are vulnerable. [10, 12] Care that patients perceive to lack genuineness can engender a sense of devaluation and isolation, while deliberately investing in GP-patient relationships can foster a sense of safety and security amidst some of the most difficult times of patients’ lives. However, we found that other attachment characteristics such as particularity, separation protest, stronger/wiser representations, strong feelings and mental representation were present in a more nuanced and less intense manner than traditional attachment relationships [18]. Specifically, while patients displayed a degree of particularity (they preferred to see their usual GP, especially regarding sensitive issues), they were often content to see another GP if theirs was unavailable. Patients expressed appreciation for their GP, and some expressed strong negative feelings towards previous GPs who they experienced as dismissive or judgemental, however this strength of feeling did not approach that of traditional attachment relationships. Patients did not express a sense of marked separation protest from their GP, although several expressed concern about what they would do when their GP retired. The knowledge differential often inherent in the GP-patient dynamic led to a nuanced ‘stronger/wiser’ representation in some patients’ minds, however patients were often also aware of GPs’ fallibility. One patient reported feeling comforted by remembering their GPs’ advice amidst mental health challenges; this was the only instance noted that may approach mental representation.

Our findings of the importance of ‘human connection’ are also consistent to some extent with Gelso’s concept of ‘real relationship’ [30]. Gelso proposed the concept of ‘real relationship’ in the context of therapist-client relationships, positing that such relationships included a ‘personal’ bond (in contrast to only a ‘working’ bond), which was characterised by genuineness and realistic (non-transference) perceptions of the other. [30] However, this concept seems to overlook the essential importance of the ‘professional’ aspect of the deep GP-patient relationship. Thus, the psychological relational models of attachment and of ‘real relationship’ appear to offer useful insights into deep GP-patient relationships, though with limitations.

The tension we found between the ‘professional’ and the ‘human connection’ was particularly striking and interesting. Human connection fostered a context that embraced GPs’ and patients’ shared humanity. GPs found providing relationship-based care both rewarding and costly, particularly when it involved going ‘above and beyond’ expectations to help patients in crises. Boundaries appear to be vital in protecting patient and GP wellbeing. Boundaries intended to protect patients from abuses of power are widely accepted. [34] However, those intended to maintain GP wellbeing or prevent unhelpful relational dependency are less well defined. [35] For actions intended to benefit patients, including those perceived to be ‘above and beyond’, the limits of professional responsibility and boundaries may be uncertain and contextual. GP participants’ individual approaches varied, consistent with the view that boundaries are not ‘black and white’ but rather fluid, and dependent upon context and individual personality and circumstances. [36] Value-informed ‘integrative wisdom’, a defining feature of generalist care, is likely to be helpful in addressing this space through its tolerance of uncertainty, awareness of complexity and ability to integrate dynamic and diverse forms of knowledge to inform practice. [37].

We found that practice staff, patients, and GPs all have a role in cultivating deep relationships. Research increasingly points to patients having an active role in general practice consultations, though perhaps due to the role of GP as care provider, patients’ relational role is rarely discussed. [38, 39] These findings suggest that patients cultivate deep GP-patient relationship by respecting their GP’s needs and boundaries, seeking continuity where feasible and investing in their own health. Moreover, findings suggest an important role for practices in relational cultivation. This aligns with previous research showing that communication with patients was influenced as much by practice policies and reception staff as by the GP themselves. [40] Our findings suggest that cohesion between practice and GP was variable. Fostering this cohesion is likely to become increasingly important with current moves toward team-based general practice primary care. [41, 42] Alongside this, the importance of long term continuity in cultivating deep GP-patient relationships suggests strategies that identify patients’ primary GP (such as voluntary patient enrolment [43]) may support these vital relationships.

Our findings, together with previous research, suggest that deep GP-patient relationships are fostered by and promote interpersonal continuity, which is associated with improved health outcomes. [44, 45] The trust underpinning such relationships assists patients to share sensitive information, supports treatment concordance, and fosters a sense of safety and security, particularly for patients experiencing vulnerability. [2, 3, 46] In the short term, such relationships are likely to come at a cost of time, though it is possible that higher quality care may save time in the longer term. [46] It is important to note, however, that GPs are unlikely to have capacity to constantly maintain deep relationships with all their patients, and not all patients desire deep relationship; rather, this seems more salient with increasing patient vulnerability. [10] Ethically, in the interest of justice for all patients, it would be important that GPs take care not to give disproportionate attention to patients with whom they have a deep relationship. Deep relationships may also risk co-dependency, though again, our data did not suggest this as a concern; the professional aspect of the relationship was perhaps protective in this respect. Additionally, while it is plausible that the emotional labour of cultivating deep relationships could contribute to burnout in some GPs, several of our GP participants commented that they find these relationships protective against burnout.

Strengths of this study include a patient-centred approach using validated tools to identify deep relationships from the patient perspective. It included GP-patient pairs, enabling comparison of GP and patient views, and provided insight into patient and practice roles in cultivating the GP-patient relationship. Participants were demographically diverse.

Our research intentionally focused on deep GP-patient relationships, which are not representative of the whole population. Our cohort were English-speaking and came from urban practices in primarily high socioeconomic areas, with an over-representation of female patient participants. Future work should explore GP-patient relationships in more diverse settings and include the views of other members of the GP practice team. Inclusion of a greater diversity of sociodemographic settings would be particularly relevant, given participants’ comments that private billing enabled GPs to invest more time with patients, and some patients’ reflections on previous negative experiences at bulk-billing practices. These comments may suggest an economic disparity in access to relationship-based general practice care in Australia. Alternately, bulk-billing practices may have other strategies for relational development. Additionally, while the small sample was appropriate to allow depth of qualitative exploration, it limited the ability to detect possible differences between participants of different attachment orientations. These could be explored with future quantitative studies.

## Conclusions

Deep GP-patient relationships comprise intertwined ‘professional’ and ‘human’ interpersonal aspects and are permeated by trust. Such relationships tend to come to the fore in difficulty, over time. They may stretch GP boundaries and capacity for self-care, but also provide joy and vocational satisfaction. Findings offer a framework to conceptualise deep GP-patient relationships and highlight the importance of creating and preserving contexts that support such relationships, particularly for patients experiencing difficulty. Deep GP-patient relationships require GPs to balance professional responsibility, interpersonal care, boundaries and wellbeing. Whole of practice cohesion is likely to be increasingly important in supporting deep GP-patient relationships as practices move toward team-based primary care. [41, 42] Findings also emphasise the importance of adequate general practice funding, enabling time to provide this vital relationship-based care.

### Electronic supplementary material

Below is the link to the electronic supplementary material.

Supplementary Material 1: Initial patient survey

Supplementary Material 2: Semi-structured interview schedules

Supplementary Material 3: Post-interview surveys

## Data Availability

De-identified datasets used and/or analysed during the current study are available from the corresponding author on reasonable request, subject to appropriate ethical approvals.

## List of abbreviations

ECR-M16: Modified and Brief Experiences in Close Relationships Scale
GP: General Practitioner
HCP-AF: Health Care Provider Attachment Figure Survey
N/A: Not applicable
PCPCM: Person-Centred Primary Care Measure
PDDR: Patient-Doctor Depth of Relationship Scale
