# Supplementary material for: Where the joy comes from: a qualitative exploration of deep GP-patient relationships
Source: BMC Prim Care. 2023 Dec 13;24:268. doi: 10.1186/s12875-023-02224-0 (PMC10717859; doi:10.1186/s12875-023-02224-0)
Supplement: Supplementary file 1 — Supplementary Material 1: Initial patient survey [file 12875_2023_2224_MOESM1_ESM.docx]

**Supplement 1: Initial Patient Questionnaire**

What is your age?

- Under 18
- 18 - 24
- 25 - 34
- 35 - 44
- 45 - 54
- 55 - 64
- 65 - 74
- 75 - 84
- 85 or older

What is your gender?

- Male
- Female
- Non-binary / third gender
- Prefer not to say

Which GP are you seeing today?

How long have you been seeing this GP for?

- Less than a year
- 1-2 years
- 2-5 years
- 5-10 years
- 10-20 years
- More than 20 years

Including your visit today, about how many times have you seen this GP in the past 12 months?

- 1-2
- 3-7
- 7-10
- More than 10

Do you currently live with a long term (6 months or more) illness or mental health condition?

- Yes
- No

Thinking about the GP who you are seeing today, please respond to the following questions:
 
(Reference: Maunder, R. G. and J. J. Hunter (2016). "Can patients be 'attached' to healthcare providers? An observational study to measure attachment phenomena in patient-provider relationships." BMJ Open 6(5): e011068.)

|  | Answer | |
| --- | --- | --- |
|  | No | Yes |
| My time with this GP is important to my wellbeing |  |  |
| This GP might see me at a time when I am in pain or feel worried, anxious or upset |  |  |
| This GP is a person I count on for advice |  |  |
| In some circumstances, I might count on this GP to help me feel better |  |  |
| This GP makes me feel more confident about my health |  |  |

*For patients who scored 4 or 5/5 on the Health Care Provider Attachment Figure Survey:*

Thinking about the GP you are seeing today, please answer the following questions as honestly as possible by selecting the option that best fits your opinion:
 
(Reference: Ridd, M. J., et al. (2011). "Patient-doctor depth-of-relationship scale: development and validation." Ann Fam Med 9(6): 538-545.)

|  | Disagree | Neither agree nor disagree | Slightly agree | Mostly agree | Totally agree |
| --- | --- | --- | --- | --- | --- |
| I know this doctor very well |  |  |  |  |  |
| This doctor knows me as a person |  |  |  |  |  |
| This doctor really knows how I feel about things |  |  |  |  |  |
| I know what to expect with this doctor |  |  |  |  |  |
| This doctor really cares for me |  |  |  |  |  |
| This doctor takes me seriously |  |  |  |  |  |
| This doctor accepts me the way I am |  |  |  |  |  |
| I feel totally relaxed with this doctor |  |  |  |  |  |

We plan to contact some survey respondants to participate in a 30-60 minute confidential interview with a member of the research team, regarding their relationship with their GP. Interview participants will be compensated for their time with a $50 gift voucher.

If selected, would you be willing to be contacted by the research team to participate in an interview?

- Yes
- No

Please provide your name and contact details (email, phone) below. This information will only be used if required to arrange an interview.

________________________________________________________________

Q12 Would you like to receive a copy of the published results of this study when available by email?

- Yes
- No

Q13 Please provide your name and the email address you would like the published results sent to:

________________________________________________________________
