# Supplementary material for: Where the joy comes from: a qualitative exploration of deep GP-patient relationships
Source: BMC Prim Care. 2023 Dec 13;24:268. doi: 10.1186/s12875-023-02224-0 (PMC10717859; doi:10.1186/s12875-023-02224-0)
Supplement: Supplementary file 2 — Supplementary Material 2: Semi-structured interview schedules [file 12875_2023_2224_MOESM2_ESM.docx]

**Supplement 2: Semi-Structured Interview Schedules**

*Note that questions were selected and modified iteratively throughout the process of data collection.*

**Patients**

- Could you tell me about how you got to know your GP?
  - Has your relationship with your GP changed over time? In what way?
  - Is there anything that surprised you about your GP?
- How do you feel when you see your GP?
- What do you value about your relationship with your GP?
  - Can you think of a time when knowing your GP made a big difference to you? What happened?
  - Can you think of a time when something serious happened in your life? How would you describe the relationship between you and your GP at that time?
- What makes having this type of relationship easier?
- What makes having this type of relationship harder?
  - Has there ever been a time when things didn’t go so well with your GP? What did you do?
  - Have you ever had a difference of opinion with your GP? How did that make you feel?
  - How do you feel if your GP isn’t available when you need to see them?
  - How do you feel if your GP looks disappointed in you?
- How do you look after your GP?
  - Do you think your GP gets anything out of seeing you?
- Have you had different (less positive) types of experiences with GPs previously? Can you describe these? In what way do they differ from your experience with your GP now?

**General Practitioners**

- Can you tell me briefly about your practice and your career so far?
- Tell me about how you get to know your patients?
- How would you describe your relationships with your patients?
- How would you describe your ideal relationship with patients?
- How do you cultivate relationships with your patients?
  - Does this differ depending on your patients’ relational styles?
- Why is it important to cultivate the relationship?
  - How much effort do you invest in cultivating the relationship?
  - Does cultivating the relationship ever conflict with delivering good patient care?
- What makes it easier to cultivate ideal relationships with patients?
- What makes it harder to cultivate ideal relationships with patients?
- How do you know if patients value their relationship with you?
- Interviewer to give examples of 2-3 specific patients (who have also consented to be interviewed separately) and ask the GP to describe the relationship and how they cultivated this with each one.
- How do you personally sustain cultivating these types of relationships over the long term?
