# Supplementary material for: Where the joy comes from: a qualitative exploration of deep GP-patient relationships
Source: BMC Prim Care. 2023 Dec 13;24:268. doi: 10.1186/s12875-023-02224-0 (PMC10717859; doi:10.1186/s12875-023-02224-0)
Supplement: Supplementary file 3 — Supplementary Material 3: Post-interview surveys [file 12875_2023_2224_MOESM3_ESM.docx]

**Supplement 3: Post-Interview Survey**

Part One: For General Practitioners (GPs) Only

Thank you for taking the time to complete this questionnaire. Your responses will help us to better understand your demographics and relational style.

What is your age range?

- 20-29yrs
- 30-39yrs
- 40-49yrs
- 50-59yrs
- 60-69yrs
- 70-79yrs
- >79yrs

How long have you been practising as a GP (including time as a registrar)?

- 0-5yrs
- 5-10yrs
- 10-20yrs
- >20yrs

How long have you been working at your current practice (including time as a registrar)?

- 0-1yrs
- 2-3yrs
- 4-10yrs
- 11-20yrs
- >20yrs

How many sessions (morning, afternoon or evening) do you work each week?

- 1-2
- 3-4
- 5-6
- 7-8
- 9-10
- >10

How long is your standard appointment time?

- 10min
- 15min
- 20min
- 30min
- Other (please specify)

What is your billing model?

- Bulk billing
- Private billing
- Mixed billing

Part Two: For GPs and Patients

The following statements concern how you feel in close relationships with others. In the following statements the term ‘other people’ refers to people with whom you feel close (eg. family, friends or anyone else who you feel close to).
 
Using the rating scale, indicate how much you agree or disagree with each statement.
 
(Reference: Adapted from Lo, C., et al. (2009). "Measuring attachment security in patients with advanced cancer: psychometric properties of a modified and brief Experiences in Close Relationships scale." Psychooncology 18(5): 490-499.)

|  | Strongly disagree | Somewhat disagree | Slightly disagree | Neutral | Slightly agree | Somewhat agree | Strongly agree |
| --- | --- | --- | --- | --- | --- | --- | --- |
| I get uncomfortable when other people want to be very close to me. |  |  |  |  |  |  |  |
| I worry about being abandoned. |  |  |  |  |  |  |  |
| I tell people with whom I feel close just about everything. |  |  |  |  |  |  |  |
| I need a lot of reassurance that I am loved by people with whom I feel close. |  |  |  |  |  |  |  |
| I don’t feel comfortable opening up to other people. |  |  |  |  |  |  |  |
| I worry a lot about my relationships. |  |  |  |  |  |  |  |
| I usually discuss my problems and concerns with people with whom I feel close. |  |  |  |  |  |  |  |
| I find that other people don’t want to get as close as I would like. |  |  |  |  |  |  |  |
| I try to avoid getting too close to other people. |  |  |  |  |  |  |  |
| I worry that other people won’t care about me as much as I care about them. |  |  |  |  |  |  |  |
| I don’t mind asking other people for comfort, advice, or help. |  |  |  |  |  |  |  |
| I get frustrated when other people are not around as much as I would like |  |  |  |  |  |  |  |
| I prefer not to be too close to other people |  |  |  |  |  |  |  |
| I worry a fair amount about losing people with whom I feel close |  |  |  |  |  |  |  |
| It helps to turn to other people in times of need |  |  |  |  |  |  |  |
| I resent it when people with whom I feel close spend time away from me |  |  |  |  |  |  |  |

Part Three: For Patients

How would you assess your primary care (general practice) experience?
Reference: Adapted from Etz, R. S., et al. (2019). "A New Comprehensive Measure of High-Value Aspects of Primary Care." Ann Fam Med 17(3): 221-230.

|  | Not at all | Somewhat | Mostly | Definitely |
| --- | --- | --- | --- | --- |
| My practice makes it easy for me to get care |  |  |  |  |
| My practice is able to provide most of my care |  |  |  |  |
| In caring for me, my doctor considers all factors that influence my health |  |  |  |  |
| My practice coordiniates the care I get from multiple places |  |  |  |  |
| My doctor or practice knows me as a person |  |  |  |  |
| My doctor and I have been through a lot together |  |  |  |  |
| My doctor or practice stands up for me |  |  |  |  |
| The care I get takes into account knowledge of my family |  |  |  |  |
| The care I get takes into account knowledge of my community |  |  |  |  |
| Over time, my practice helps me to stay healthy |  |  |  |  |
| Over time, my practice helps me to meet my goals |  |  |  |  |
